# Recent Development of Extremophilic Bacteria and Their Application in Biorefinery

**DOI:** 10.3389/fbioe.2020.00483

**Published:** 2020-06-12

**Authors:** Daochen Zhu, Wasiu Adewale Adebisi, Fiaz Ahmad, Sivasamy Sethupathy, Blessing Danso, Jianzhong Sun

**Affiliations:** ^1^Biofuels Institute, School of the Environment and Safety Engineering, Jiangsu University, Zhenjiang, China; ^2^State Key Laboratory of Applied Microbiology Southern China, Guangdong Provincial Key Laboratory of Microbial Culture Collection and Application, Guangdong Open Laboratory of Applied Microbiology, Guangdong Institute of Microbiology, Guangzhou, China

**Keywords:** biofuel, biorefinery, extremophiles, extremozymes, synthetic biology

## Abstract

The biorefining technology for biofuels and chemicals from lignocellulosic biomass has made great progress in the world. However, mobilization of laboratory research toward industrial setup needs to meet a series of criteria, including the selection of appropriate pretreatment technology, breakthrough in enzyme screening, pathway optimization, and production technology, etc. Extremophiles play an important role in biorefinery by providing novel metabolic pathways and catalytically stable/robust enzymes that are able to act as biocatalysts under harsh industrial conditions on their own. This review summarizes the potential application of thermophilic, psychrophilic alkaliphilic, acidophilic, and halophilic bacteria and extremozymes in the pretreatment, saccharification, fermentation, and lignin valorization process. Besides, the latest studies on the engineering bacteria of extremophiles using metabolic engineering and synthetic biology technologies for high-efficiency biofuel production are also introduced. Furthermore, this review explores the comprehensive application potential of extremophiles and extremozymes in biorefinery, which is partly due to their specificity and efficiency, and points out the necessity of accelerating the commercialization of extremozymes.

## Background

Extremophiles are a group of organisms that thrive under extreme environmental conditions (e.g., high/low temperature, pH, salinity, and pressure) that most life forms find it difficult to survive in. However, most of the earth's crust is covered by extreme environmental conditions in terms of temperatures that can range from −89°C in the Antarctic/Arctic regions to 400°C at the deep seafloor (doi: 10.1016/j.biotechadv.2015.11.001). It is thus not surprising that extremophiles have evolved and developed promising strategies and mechanisms to survive best in extreme conditions of pH, temperature, pressure, and other life-supporting conditions (Navanietha Krishnaraj and Sani, 2017; Singh et al., 2019b).

Extremophiles are comprised of animals, plants, insects, fungi, and bacteria. However, in this review, we only deal with extremophile bacteria with special reference to biofuel and bioenergy (if there is no special explanation, extremophile refers to extremophilic bacteria in the following text). Extremophilic bacteria could be described as acidophilic (optimally thrive at low pH), alkaliphilic (optimally thrive at high pH), halophilic (thrive well in high concentrations of salt), thermophilic (optimally thrive at high temperature), hyperthermophilic (optimally survive extreme high temperature usually above 80°C), psychrophilic (thrive well at low temperature), piezophilic/barophilic (optimized growth at high pressure), oligotrophic (grow in nutrient deficient environments), endolithic (grow within rock spaces), and xerophilic (thrives in a dry area). Besides, polyextremophiles and extremotolerant microorganisms are also present in various extreme ecological niches (Dumorné et al., 2017; Gundala and Chinthala, 2017; Singh et al., 2019b). Researchers are interested in understanding their metabolic cycles to utilize them for potential industrial use due to their high stability at elevated temperatures, favoring of the good solubility of substrates, high mass transfer rate, and their lowering of the risk of pollution during industrial processes (Chen and Jiang, 2018).

**Figure 1:**
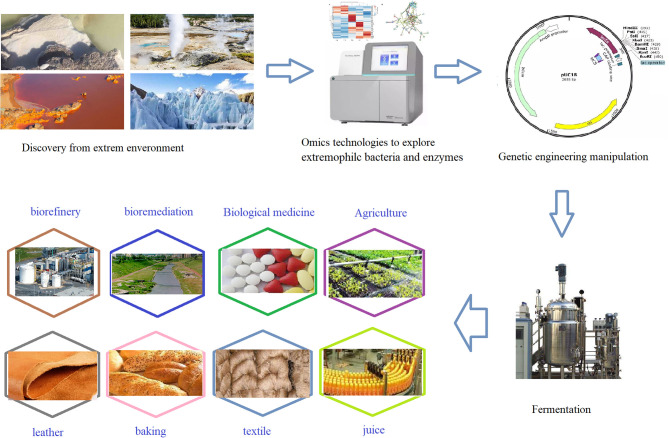
A simple flow line sketch of extremozymes production and application.

Recently, extremophiles have gained immense interest owing to their ability to catalyze reactions and potential industrial applications under severe conditions (Gupta et al., 2014; Dumorné et al., 2017; Geng et al., 2018). Though extremozymes were identified several decades ago, researchers are still concentrating more on genetic engineering of existing enzymes to potentiate their activity and the screening of novel enzymes from various sources to obtain the necessary characteristics amenable for industrial and biotechnological applications (Figure 1). Many research groups and companies around the world are committed to engineering microorganisms genetically with desirable industrial characteristics suitable for their industrial operations (Hermann et al., 2018). Many common commercial enzymes cannot meet the industrial requirements, such as being able to withstand industrial requirements with high reproducibility in different pH, temperature, and aerification conditions. Therefore, extremozymes have received increased attention as a strategy of industry process and biorefining (Espliego et al., 2018). Novozymes and Genesco have commercialized cold-adapted amylases and proteases that can eliminate starch stains (Dumorné et al., 2017). Industrial enzymes, such as those used in biorefinery, are considered as technical enzymes, and they were valued for more than $ 1 billion in 2010 and are expected to grow to $5.0 billion in 2021 at a rate of 4.0% per year (Kuila and Sharma, 2017). This was surpassed earlier, as they reached $5.5 billion in 2018 and are now projected to reach $7.0 billion by 2023 by several research associations (Freedonia, 2018; Research Biotechnology, 2018; Silveira et al., 2018). Also, a report pointed out that the global market for special enzymes (including extremozymes) reached $4.0 billion in 2018. This opens a new window for researchers to meet the ever-growing global market demand, and extremozymes are the best candidates for consideration (Dumorné et al., 2017).

The term “Bioenergy” refers to solid, liquid, and/or gaseous substances that have tended to be used as an energy source (e.g., bio-ethanol, butanol, biodiesel, and biomethane) (Fatih Demirbas, 2009; Arevalo-Gallegos et al., 2017; Moreno and Olsson, 2017). A biorefinery, a widely used concept, is a facility that permits the full, integrated use of biomass generated into a spectrum of bio-based products and bioenergy (Cherubini, 2010). Lignocellulose is the main structural component of plants that contains cellulose, hemicellulose, and lignin as its constituents. Lignocelluloses are the best candidate feedstocks for bioenergy production because of their high mass availability, relatively low price, and lack of competition with food provision (Sánchez and Cardona, 2008; Zhang Z. et al., 2016; Arevalo-Gallegos et al., 2017). However, the recalcitrance of lignocelluloses caused by lignin is the key hindrance in the utilization of this valuable resource (Moreno and Olsson, 2017; Geng et al., 2018). Therefore, the effective delignification of the biomass will play an important role in the economic feasibility of biofuel processing (Menon and Rao, 2012; Arevalo-Gallegos et al., 2017). There seems to be a bright future in the study of the delignification and utilization of the lignocellulosic biomass for bioenergy production using the most appropriate extremophilic organism (Amoozegar et al., 2019). In addition, the efficiency of hydrolysis of lignocellulose by enzyme complexes (celluloses, hemicellulose, and accessory proteins) is still a key limiting step of biorefinery (Klein-Marcuschamer et al., 2012). Therefore, it is necessary to develop an improved biorefinery cellulase that has higher catalytic efficiency, higher temperature stability, certain pH stability, and tolerance to the inhibition of the end product.

Pretreatment is the key operation unit of a biorefinery, and its purpose is to improve the accessibility of carbohydrates for biodegradation by delignification, expand the accessible surface area of biomass, and reduce the crystallinity of cellulose (Kumari and Singh, 2018). Extremophiles and their ligninolytic enzymatic systems have been used as single biological pre-treatment methods or combined with other pre-treatment technologies to improve the hydrolysis performance of biomass (Ragauskas et al., 2006; Moreno et al., 2015). The process of bioenergy production depends on the utilization of severe reaction conditions, such as high temperature or low temperature and extremes in pH and salinity. The cost of these biotechnology applications can be reduced by using microorganisms with special capabilities, and extremophiles and their enzymes would thus provide a better choice for biofuel production. In this context, this review is essentially focused on the past and current situation of extremophiles and extremozymes application in biorefineries as well as the potential technical methods for the production of low-cost bioenergy from biomass in the foreseeable future.

## Extremophiles and Extremozymes

Many extremophiles and enzymes have been isolated and have seen attempted use for biomass processing, including genus *Acidithiobacillus, Arthrobacter, Bacillus, Caldicellulosiruptor, Clostridium, Coprothermobacter, Enterobacter, Geobacillus, Micrococcus, Paenibacillus, Penicillium, Picrophilus, Pseudoalteromonas*, and *Thermobifida*. The enzymes they secreted include α-amylase, subtilase, β-galactosidase, xylanase, β-glucosidase, decarboxylase, endoglucanase, dehydrogenase, tetrathionate hydrolase, etc. These enzymes and extremophiles are summarized in Table 1.

**Table 1:** Applications of extremophiles and extremozymes in industry.

**Species**	**Source**	**Enzyme**	**Optimum reaction temperature (°C)**	**Use (potential)**	**References**
*Bacillus mojavensis* SO-10	No information	α-amylase	70	Biorefinery, food, detergent	Ozdemir et al., 2018
Thermophilic *Anoxybacillus* sp. GXS-BL	Hot spring	α-amylase	60	Food, pharmaceutics, textile, detergent and bioenergy industries	Liao et al., 2019
*Anoxybacillus thermarum* FRM-RBK02	Hot spring	α-amylase	80	Biorefinery, food, detergent	Mantiri et al., 2019
*Erwinia* sp. E602	Frozen soil	Cold-adapted β-galactosidase	40	Dairy industry	Xia et al., 2018
*Anoxybacillus flavithermus Bacillus licheniformis*	Hot spring	β-galactosidase	60	Food, bioremediation, biosensor	Rani et al., 2019
*Alteromonas* sp. ML117	Marine	β-galactosidase	10	Food	Yao et al., 2019
*Bacillus tequilensis* ARMATI	Feces soil	Thermo-alkali stable xylanase	60	Biorefinery, food	Khusro et al., 2016
*Bacillus subtilis* Lucky9	No information	Alkali tolerant xylanase	60	Biofuel and food	Chang et al., 2017
*Dictyoglomus turgidum*	No information	Thermostable β-glucosidase	80	Biorefinery, food	Fusco et al., 2018
*Exiguobacterium antarcticum* B7	Antarctica soil	Cold-adapted β-glucosidase	30	Biorefinery, ethanol	Crespim et al., 2016
*Micrococcus antarcticus*	Antarctica	Psychrophilic β-glucosidase	35	Detergents, textiles, bioremediation	Miao et al., 2016
Metagenome	Marine	Thermoactive endoglucanase	115	Biorefinery	Suleiman et al., 2019
*Cellulomonas fimi* ATCC484	Soil	Thermostable endoglucanase	65	Biorefinery	Saxena et al., 2018
*Bacillus cereus* FT 1	Soil	Alkaline protease	35	Detergents, pharmaceutical, leather, food, bioremediation	Asha and Palaniswamy, 2018
*Janibacter* sp. strain R02	Antarctic soil	Thermophilic and halophilic esterase	80	Detergents, pharmaceutical, leather, food, bioremediation	Castilla et al., 2017
Thermophilic *Anoxybacillus* sp. HBB16	Hot spring	Alkaline lipase	50	Organic synthesis, detergent, wastewater treatment, biodiesel	Burcu Bakir and Metin, 2017
*Paenicibacillus barengoltzii*	Marine	Chitinase	55	Conversion of cellulose to ethanol	Yang et al., 2016

### Thermophiles and Thermostable Enzymes

Thermophiles are organisms that thrive at 41–122°C with optimum growth temperatures between 60 and 108°C. These organisms have attracted great attention among extremophiles due to their being the main source of industrially important thermostable enzymes (Singh et al., 2011). Thermophiles can generally be divided into moderate thermophiles (T_opt_: 50–60°C), extreme thermophiles (T_opt_: 60–80°C), and hyperthermophiles (T_opt_: 80–110°C) (Singh et al., 2011). For example, it has been reported that the hyperthermophiles *Methanopyrus kandleri* 116 can even grow at 122°C, which has broad application prospects in industrial processes (Su et al., 2013).

The use of thermostable enzymes in the biorefining process gives substrates properties of better solubility, easier mixing, having a reduced the risk of contamination, and higher conversion efficiency. Thermophilic bacteria are often considered one of the sources of industrially relevant thermostable enzymes (Rigoldi et al., 2018). Generally, an enzyme or protein is considered to be thermostable when it has a high defined unfolding (transition) temperature (Tm) or a long half-life at a selected high temperature (Böhme et al., 2019). These unique characteristics of thermostable enzymes pave a way for their widespread applications in industries. For example, thermostable enzymes obtained from thermophilic bacilli have found a plethora of commercial applications due to their robustness in catalytic activity and their ability to withstand the heat generated in various biotechnological and industrial processes (Margaryan et al., 2018). Thus, thermophilic enzymes can be used to catalyze high-temperature chemical processes that are difficult for normal-temperature enzymes. By far the most successful extremozyme is “Taq polymerase,” which was isolated by F.C. Lowyer from thermophilic bacteria “*Thermus aquaticus*.” Similarly, thermophilic *Geobacillus* spp. and *Geobacillus* sp. Iso5 were isolated, characterized from thermal springs, and were shown to produce hyper thermostable α-amylase with an optimum enzyme activity at 90 and 140°C, respectivel; they are thus extremely promising for biorefinery application (Nazina et al., 2000, 2001; Gurumurthy and Neelagund, 2012).

It has been reported that a variety of thermophilic bacteria and their secreted enzymes have great application potential in bioenergy production and anaerobic fermentation, and these include *Caldicellulosiruptor bescii, Geobacillus proteiniphilus, Thermoanaerobacterium, Pyrococcus*, and *Caldicellulosiruptor* (Jiang et al., 2018; Williams-Rhaesa et al., 2018; Semenova et al., 2019; Straub et al., 2019; Hoffmann et al., 2020). Thermophilic *Clostridium* has been successfully used as green biologics to produce biobutanol using corn stock as a substrate (Zhang and Jia, 2018). The inoculation of *Consortium* TC-5 in digested sludge was reported to significantly increase the methane production rate by 36.6% under thermophilic conditions, which is conducive to the sustainable development of the industrial economy (Kong, 2018). Most recently, a glycocin biosynthetic gene cluster from a thermophilic *Aeribacillus pallidus* 8 was expressed heterologously in *E. coli* as a good tool for the production of hypothetical glycocin, which holds the potential application in biofuel production (Arnoldas Kaunietis et al., 2019). In a recent study, a butanol-producing *Clostridium* sp. strain WST was introduced *via* the bioaugmentation process, which significantly improved the butanol yield and up to 0.54 g/g by 98-fold. This breakthrough offers an eco-friendly way to efficiently use lignocellulose to produce biofuels and bioenergy, which in turn reduces the production cost (Shanmugam et al., 2019). Generally, thermophiles and their enzymes/proteins have potential usage in biomass pretreatment and biofuels production. However, the disadvantages of using thermophilic bacteria to produce biofuels include the lack of both scientific understanding of thermophilic bacteria and of appropriate genetic manipulation tools. Recently, an efficient and rapid gene knock-out/knock-in system was established by using the thermosensitive replicon and a heterologous pyrE gene of *Geobacillus kaustophilus* as the counter-selection marker, which enabled its use in thermophilic bacilli as a considerable improved metabolic engineering tool (Sheng et al., 2017). Another challenge is that a biorefinery needs a whole enzyme platform, and it is difficult for a single thermostable enzyme to integrate into the conventional enzyme system. A single enzyme will lead to a synergistic effect and affect the application of the enzyme system in biorefineries (Broeker et al., 2018). Therefore, building a whole enzyme platform of the thermostable enzyme system may be one of the important research directions in the future. The current challenge is the construction of chassis cells using a synthetic biology approach and the integration of extremophile-derived enzymes to maximize their industrial application.

### Psychrophiles and Psychrotolerant Bacteria

So-called psychrophilic or psychrotolerant organisms are usually found in niche, low-temperature areas of the world, including the Antarctic or Arctic, freezing appliances, glaciers, shallow underground areas, deep oceans, high-altitude atmospheres, and so on or in plants and animals living in cold regions (Cavicchioli et al., 2009). According to Gounot, cold-adapted bacteria can be classified as psychrophilic (permanently cold) and psychrotrophic (seasonally cold or where temperature fluxes into mesophilic range) (Gounot, 1986). Variations in the soil microbial community structure of mountainous regions have been shown to be associated with altitude level, and the relative abundance and diversity of bacteria were reported to be decreased with an increase in altitude level (Zhang et al., 2009; Margesin and Miteva, 2010; Martin and McMinn, 2017). In the marine ecosystem, psychrophilic bacteria are dominant due to prevailing temperatures (<5°C); the deep sea and most of the cold-adapted microbes isolated from the fluctuating land environment are considered to be psychrotolerant (Bölter, 2004).

In recent years, numerous psychrophilic microorganisms with promising industrial application have been isolated and identified from various extreme environments. A novel psychrotolerant *Sanguibacter gelidistatuariae* was isolated from an ice sculpture in Antarctica, and it was found to grow at 3°C (Pikuta et al., 2017). An alkaliphilic and psychrotolerant novel species *Carnobacterium antarcticum* CP1 was isolated from sandy soil near the Davis Station in Antarctica (Zhu S. et al., 2018). Appropriate genetic manipulation tools are very important for the application of psychrophilic bacteria. Four plasmids were identified in *Psychrobacter* sp. DAB_AL43B, and they can be developed as genetic manipulation tools for the construction of *Escherichia col*i-*Psychrobacter* spp. shuttle vectors (Lasek et al., 2017). The exopolysaccharides (EPS) were synthesized as a strategy of psychrophilic bacteria to resist the extreme conditions; they can be used in biomedicine and the food industry as well as for biomaterials, casting, and electrospinning. However, although many biological processes have been developed to produce EPS, the efficiency of EPS production by extremophiles is very low compared with medium and moderate neutral EPS-production bacteria (De Carvalho, 2019). How to use synthetic biology to develop metabolic and genetic engineering strategies for the enhancing yield of EPS is of great significance to the elimination of obstacles in the industrialization of novel EPS.

The “cold-adaptive” enzymes, termed psychrozymes, can thrive at very low temperatures (typically below 15°C). Their cold-activity characteristics give them many advantages in biotechnology due to the high Kcat at low to moderate temperatures in biofuels and energy production (Wierzbicka-Woś et al., 2011; Martin and McMinn, 2017). The advantage of psychrophilic bacteria and their cold-adapted enzymes is that they can treat and digest lignocellulose at low temperatures. This greatly reduces the energy input required to heat the bioreactor and avoids chemical side-effects that can occur at higher temperatures and the generation of adverse byproducts. In addition, the mild temperatures of the industrial conditions were better for preventing the denaturation of thermosensitive substrates. Mild temperature will make the industrial operation more convenient and safer, which is the developmental trend of traditional industry (Hamid et al., 2014). Moreover, cold-active enzymes have advantage for the *in-situ* bioremediation or bioaugmentation of chemical or oil-contaminated cold environments (Alpine soils, the temperate zone, and the frigid zone) and cold-adapted cellulose-degrading enzymes have practical significance for solving the problem of slow decomposition of straw residue in the straw incorporation (Kavitha, 2016). Many psychrozymes have been isolated from the psychrophiles of deep sea and the polar regions, such as amylase, protease, lipase, pectinase, xylanase, cellulase, β-galactosidase, β-glucosidase, chitinase, etc. These can find potenitial application on an industrial scale (Pooja et al., 2009; Feller, 2013; Siddiqui, 2015). Cold-active endoglucanase (CelX) from psychrotrophic *Pseudoalteromonas* sp. DY3 and β-galactosidase enzyme produced by *Enterobacter ludwigii* are regarded as possible and economically attractive alternatives to hydrolyze lignocellulose at refrigerated temperatures (Zeng et al., 2006; Alikkunju et al., 2016; Alikunju et al., 2018). These psychrophilic endocellulases can also be used as detergents in laundry as well as to produce biofuels and chemicals (Yuan et al., 2018). Benefits from the latest advances in synthetic biology, metabolic engineering, X-ray crystallography, structural modeling, protein engineerings and biophysical research facilitates the identification, characterizations and optimization of reaction conditions of new psychrozymes from psychrophiles for biomass treatment and bioenergy production.

### Alkaliphiles and Acidophiles

Alkaliphiles are a class of extremophiles that survive in alkaline environments (i.e., pH 8.5–11 with optimal growth at pH 9), such as in soda lakes, hydrothermal vents, hind-gut of insects, deep-sea sediments, and carbonate-rich soils (Preiss et al., 2015). Conversely, acidophiles or acidophilic organisms tend to grow well in acidic conditions (i.e., pH 1–5 with optimum growth below pH 3), and these are mainly present in sulfuric pools, solfataric fields, and coal and sulfur mines (Johnson and Schippers, 2017).

Enzymes from alkaliphiles with high thermostability, alkaline activity, and substrate specificity have added more advantage for biofuels production under harsh industrial conditions (Annamalai et al., 2016). Published in 1971, the first report concerning alkaline enzyme was an alkaline cellulase produced by *Bacillus* species (Horikoshi et al., 2011). An alkaliphilic *Bacillus ligniniphilus* L1 was isolated from the South China Sea. It grows well at 30°C and a pH of 9.0, and it has a potential application value in the lignin valorizations (Zhu et al., 2013; Zhu D. et al., 2017). A glycoside hydrolase (GH5)-encoding gene from *Thermobifida halotolerans* YIM 90462^T^ was expressed in *E.coli*, and its biochemical characterization showed it could be a good candidate enzyme for the application of cellulose degradation (Zhang et al., 2015). In addition, alkaliphilic xylanase was used in the bleaching process of kraft and soda pulps without adjusting the pH, which improved the economic feasibility (Weerachavangkul et al., 2012). It is more economical and energy saving to use alkaline hemicellulase in hemicellulose biorefinery because hemicellulose has better solubility and higher hydrolysis efficiency at high pH, and the direct enzymatic hydrolysis of alkali extracted hemicellulose to monomeric sugars at high pH avoided time-consuming and corrosive pH regulation (Mamo, 2019). In order to meet the demands of a biorefinery, it is an important research goal to improve the pH and thermostability of alkaline xylanase by site-directed mutation and directed evolution (Bai et al., 2016; Li et al., 2019; Xiang et al., 2019).

The unique structures and functions of acidophilic bacteria, such as membrane potential reversal, high membrane impermeability, and the presence of secondary transporters, make them have a broad application prospect in the biological industry. A xylanase produced by acidophilic bacteria *Penicillium oxalicum* GZ-2 has been shown to be useful in biofuels, animal feed, and food industries (Liao et al., 2012), and, more importantly, utilization of acid-tolerant xylanase pulp bleaching at low pH levels is more advantageous than normal processes (Michaux et al., 2010). An Acidophilic *Stenotrophomonas maltophilia* was isolated and was found to be useful in biodegradation of polycyclic aromatic hydrocarbons (Patel et al., 2005; Arulazhagan et al., 2017).

### Halophiles and Halotolerant

Halophiles are extremophilic microorganisms that can grow optimally in saline and hypersaline environments, such as deep-sea sediments, saline lakes, salt pans, saline soils, and sea water (Kumar and Khare, 2012), whereas halotolerant microbes are able to survive in high-salt environments and also grow well under normal conditions. These inherent characteristics of halophilic and halotolerant microorganisms and the demand for salt tolerance depends on environmental and nutritional factors. They are therefore one of the best choices for biofuel production and other industrial processes (Singh et al., 2019c). It is worth mentioning that the enzymes for biofuel production, such as cellulase, xylanase, laccase etc., from halophilic and halotolerant microorganisms are relatively more stable than those of a terrestrial origin. It has been reported that a halotolerant *Haloarcula* sp. Strain LLSG7 with high cellulolytic activity from the saline soil of Yuncheng Salt Lake, China, produced five different extracellular endoglucanases with an outstanding stability in the presence of organic solvents. The enzymatic hydrolysate of crude cellulase produced by strain LLSG7 was used as a substrate for bioethanol fermentation by *S. cerevisiae* yielded 10.7 g/L of ethanol, which was much higher when compared with those reported from other cellulases (Li and Yu, 2013). In addition, the polyextremotolerant cellulases from *Paenibacillus tarimensis* L88 showed its optimum activity at 80°C and broad pH range (3.0 to 10.5) in the presence of a high salt concentration (Raddadi et al., 2013). A halophilic bacterium *Nesterenkonia* sp. F can tolerate organic solvents and produce α-amylase, and it is the only wild bacterium that can produce butanol and ethanol except *Clostridia* (Amiri et al., 2016). Many enzymes, such as α-amylase, β-amylase, glucoamylase, lipase, esterase, hemicellulose, and ligninase of a halophilic and halotolerant bacterial origin hold potential industrial applications, such as bioethanol, biodiesel, or fatty acids production using renewable resources (Schreck and Grunden, 2014; Elmansy et al., 2018; Amoozegar et al., 2019). Moreover, in order to improve the performance of halophilic bacteria for industrial application, researchers are currently committed to developing various genetic tools and using synthetic biology and genetic modification technology to accelerate cell growth and enzyme production (Chen and Jiang, 2018). Through PCR-based site-saturation mutagenesis, the activity of mutant halophilic α-amylase (threonine was replaced by aspartic acid) was increased 14.6 times compared with that of natural enzyme under salt-free conditions (Pan et al., 2020).

### Defense Mechanism of Extremophiles Under Various Conditions

**Figure 2:**
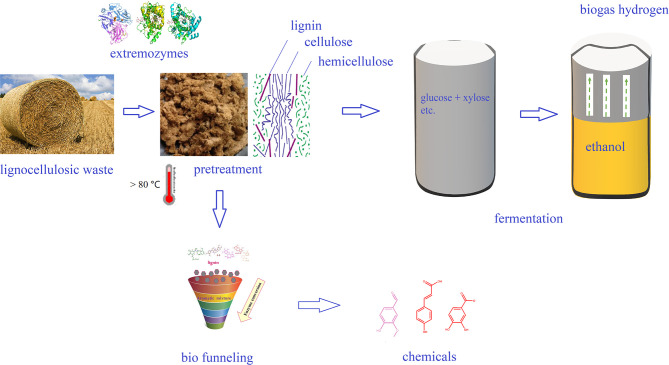
Application of extremozymes in bioenergy and biochemicals production.

In order to survive in the adverse environment, extremophiles have developed a variety of strategies to cope with the harsh environment (Figure 2). The heat above the physiological temperature readily lead to the unfolding and denaturation of proteins, as it destroys the intracellular bonds that are harmful to organisms. Thermophilic microorganisms are able to restore their protein structure and function by producing chaperones or thermosomes under extreme condition to resist the destruction of protein by high temperature (Annamalai et al., 2016). In order to resist the protein unfolding caused by high temperature, thermophilic bacteria have developed special hydrogen bonds that can interact with hydrophobicity. Meanwhile, thermophilic bacterial enzymes are rich in salt bridges and/or extra disulfide bridges to make its structure more stable (Chakravorty et al., 2017). In addition, other thermo-resistance factors, including structural compactness, oligomerization, glycosylation, and hydrophobic interactions between subunits, are crucial for stability (Chakravorty et al., 2017). Synthetic biology, including site-directed mutagenesis and directed evolution of enzymes, has been used to improve the thermal stability of target proteins/enzymes, which is essential for their application in bioenergy industry (Adesioye et al., 2018).

In contrast to thermophiles, psychrophiles are able to live in extreme cold conditions, and this is mainly due to their cellular cold-adaptability mechanisms: the regulation of cold-shock proteins, small RNA-binding proteins, and extracellular polymeric substances to protect the cells against mechanical disruption to the cell membrane caused exerted by low temperature. In addition, the genome of psychrophiles contain higher G+C-rich regions encoding tRNAs, elongation factors, and RNA polymerases, and the presence of plasmids, transposable/mobile genetic elements related to the biosynthesis of unsaturated fatty acids improves their cold adaptability. Furthermore, psychrophiles with high translational and post-translational processing capacity may be essential for their growth at low temperatures (De Maayer et al., 2014). In order to adapt to cold environment, psychrophilic enzymes usually have higher structural flexibility, lower thermal stability, and higher specific activity.

Acidophiles use a variety of homeostatic pH mechanisms that involve restricting/passive proton entry into the cytoplasmic membrane and purging off protons (Mirete et al., 2017). They also have a highly impermeable cell membrane to restrict the proton influx into the cytoplasm by active proton pumping (Zhang X. et al., 2016). The well-studied *Picrophilus oshinae*is is a typical example that is capable of thriving at pH 0.7 despite its internal pH being 5 (Madigan, 2000). In alkaliphiles like *Natronobacterium gregoryi*, for example, the ions are pumped inside the cells to maintain the cytoplasmic pH at near neutrality (Abe and Horikoshi, 2000).

In order to survive in the high-saline environment, halophiles developed strategies to keep the osmotic balance between intracellular with the environment to prevent water loss. One such strategy is the intracellular synthesis or accumulation of compatible solutes/osmolytes, such as ectoine, trehalose, proline, dimethylsulfoniopropionate K-glutamate, betaine, and carnitine (Zhu et al., 2008; Vauclare et al., 2014). For example, ectoine-mediated homeostasis maintenance mechanism enables halophiles to withstand and grown in the hypersaline environment (Zhu et al., 2010; Babu et al., 2015). Halophile-derived solutes such as ectoine are used in bio-industry for biofuel production. Supplementation of ectoine in the growth medium of *Zymomonas mobilis* has been shown to improve the ethanol production (Zhang et al., 2008).

### Challenges of Application of Extremophiles and Extremozymes in Biorefinery

Despite the fascinating prospect of extremophiles and extremozymes, there are still obstacles in large-scale industrial application. It is difficult to cultivate extremophiles on a large scale due to harsh culture conditions. For example, using halophilic bacteria to produce Polyhydroxylalkanoate (PHA) requires a high-salt medium, but high salt concentration resulted in frequent and expensive maintenance of equipment. In addition, the process of extracting PHA from a high-salt culture is complex and costly, and the treatment of high-salinity wastewater is also a challenge (Zhang et al., 2018; Liu et al., 2019). The lack of suitable genetic tools makes it difficult to improve the efficiency of hydrolysis and increase the product yield of extremophiles by metabolic engineering. It is still difficult for the genetic manipulation of *Halomonas*, the suicide plasmid-mediated homologous recombination system is a time-consuming, low-efficiency method for the engineering of *Halomonas bluephagenesis* TD01 (Fu et al., 2014). Although many extremophiles were culturable, the number was still very tiny compared to uncultured extremophiles. At present, most of the extremophiles were difficult to isolate and identify by the existing culture methods. It was recently estimated that the average number of uncultured microorganisms in the genus level was 7.3 × 10^29^, of which 81% of the microbial cells were distributed in terrestrial, underground, and high-salt environments as well as marine sediments, hot springs, and hydrothermal vents (Lloyd et al., 2018). The huge technological gap between producing enzymes in laboratory conditions and obtaining the final commercial product is a challenge when developing extremozymes. It can be seen that a large number of extremozyme-related papers are published every year, but they rarely achieved industrialization (Di Donato et al., 2018; Jin et al., 2019; Varrella et al., 2020). In recent years, more and more scientists have devoted themselves to these issues affecting the application of extremophiles in the field of biorefinery, and they have obtained many achievements. The CRISPR/Cas9 system was developed for the genome editing of extremophiles and is expected to be used for metabolic engineering applications (Mougiakos et al., 2017). Schiraldi et al. designed a bioreactor for the production of β-glucosidase from thermophilic *Sulfolobus solfataricus* based on a microfiltration hollow-fiber module inside of a traditional bioreactor; it enhanced the cell density from 2 to 35 g/L (Schiraldi et al., 1999). In order to solve the corrosion issue of high salt concentrations to the bioreactor, new materials were developed to replace all of the stainless-steel parts of bioreactor and autoclavable materials, such as Polyether ether ketone (Pandey et al., 2017).

## Potential Industrial Applications of Extremozymes

Extremozymes as biocatalysts possess extraordinary properties, such as thermostability, cold adaptivity, and osmotic allowance, to permit them using in biorefinery, agriculture, chemical industry, bioremediation, biomedicine, and environment pollution control. In this part, we focus on the potential of extreme enzymes in lignocellulose treatment, biofuel production, and high-value utilization of lignin.

### Pretreatment of Lignocellulosic Biomass With Extremozymes

Lignocellulosic raw materials come from a wide range of natural sources and are considered an abundant renewable resource for the production of biofuels and value-added chemicals. In recent years, the preparation of fuel ethanol from lignocellulose has attracted much attention due to its eco-friendly nature (Zabed et al., 2016). The development of lignocellulosic energy technology is a “win-win” model for reducing costs and protecting the environment, which is conducive for the sustainable development of biorefinery based economy (Birch, 2019). At present, the cost of biofuel production from plant fiber is still high, and it has thus turned out to be one of the research hotspots to ease the process of biofuel preparation as well as to develop the cost-effective pretreatment method. In recent years, enzymes from microbial origin play a leading role in the biofuel production. When compared with chemical methods, the application of enzymes in industrial bioprocesses reduces the risk of pollution, hence they are therefore considered to be better substitutes for lignocellulose pretreatment (Ummalyma et al., 2019). Owing to the stability and robust catalytic activity of extremozymes, further development of methods involving extreme pH and temperature conditions is much needed research input to accelerate the industrial processes.

Lignocellulose is decomposed into three different polymers, such as lignin, hemicellulose, and cellulose by pretreatment, and cellulose is then converted into a monosaccharide by cellulase. Biofuel (bioethanol) is then produced by fermentation. The acid- and alkali-tolerant nature of extremozymes are helpful for pretreatment as well as the complete hydrolysis of cellulose/hemicellulose at high temperature. Lignocellulose hydrolase is limited by many factors, including crystallinity and polymerization degree, water expansion, water content, surface area, and lignin content. Lignocellulose pretreatment at industrial scale should be potent enough to overcome the complex physicochemical, structural, and compositional barriers that inhibit the biomass digestion and improve the hydrolysis rate of lignocellulosic materials (Karimi and Taherzadeh, 2016). Hence, the combination of different pretreatment technologies are currently being employed, for example, a hydrothermal-assisted enzyme degradation process is one of the effective and widely used pretreatment methods (Kirsch et al., 2011). The advantages of this combination of multiple pretreatment processes include energy saving by omitting the cooling step and better operability at high temperatures, such as improving the accessibility of the substrate while reducing viscosity (Bhalla et al., 2013).

Biological pretreatment method involving laccase, along with other pretreatment methods, have been reported to significantly improve the delignification of lignocellulose (Moreno et al., 2015). During removal of lignin from biomass using chemical process, hemicellulose and cellulose can be partially degraded. However, biological pretreatment of biomass with extremophiles and lignin decomposition enzymes reduces the amount of the degradation products as well as time required for the pretreatment. In addition, the combination of chemical and biological pretreatment processes of eucalyptus, wheat straw, pine, and corn straw have increased the delignification efficiency (Moreno et al., 2015). Meanwhile, the high cost of enzymes makes it the most skilled but also the most expensive means in the second generation of biorefinery concept. How to further reduce the production cost of extreme enzymes and improve their activity and stability is a challenge. Further research is needed to break these obstacles and improve the pretreatment methods, and the delignification of extreme enzymes is an amazing and attractive choice for the future biorefinery.

### Utilization of Extremophiles and Enzymes in Liquid Biofuels Production

**Figure 3:**
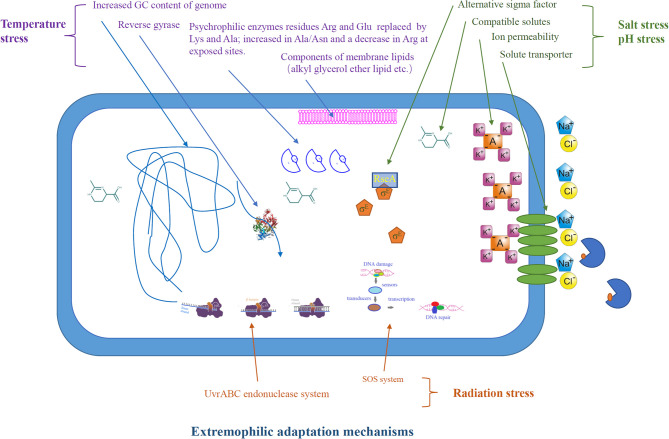
Extremophilic adaptation mechanisms of extremophiles in response to environmental stress.

Among all the biofuels, bioethanol is often regarded as the most promising alternative and/or additive for gasoline. In China, gasoline stations in many provinces utilize ethanol as an additive. The production of bioethanol from lignocellulosic biomass, named “second generation bioethanol,” includes four main steps: biomass pretreatment, enzymatic hydrolysis, fermentation, and distillation (Indira et al., 2018). The production of bioethanol from lignocellulosic raw materials is beneficial to environment and helps sustainable development, but the second-generation technology still have problems of high cost, and there are several areas of production technology that still require improvement to cut down the cost. Because of the unique characteristics, extremophiles are resistant to the adverse conditions involved in bioethanol production, and they thus harbor more advantages than terrestrial microorganisms. In particular, thermophiles and their enzymes have great potential for the bioconversion of lignocellulose into bioethanol (Figure 3). Certain thermophilic bacteria are known to produce both cellulase and xylanase, which can completely hydrolyze biomass at high temperature. For example, treatment of biomass using a thermostable cellulase produced by thermophilic *Geobililus* sp. R7 has been shown to yield a hydrolysate that was readily fermented by *Saccharomyces cerevisiae* ATCC 24860^T^ to produce 0.45–0.50 g ethanol/g glucose with a 99% utilization rate of glucose (Zambare et al., 2011). Thermophilic *Caldicellulosiruptor bescii* and *Clostridium thermocellum* have been reported for its potential to use cellulose, hemp, as well as pretreated lignocellulosic biomass as a substrate to yield bioethanol (Olson et al., 2012). In addition, thermophiles *Thermoanaerobacterium thermosaccharolyticum* M18 is able to directly utilize cellulose and xylan for the production of bioethanol (Ábrego et al., 2017).

Although thermophilic bacteria have many advantages and utilize a broad spectrum of degradable carbohydrates and the fermentation of hexose and pentose and offer a low risk of pollution, the problems associated with the low G+C content, the formation of endospores, and the low permeability of plasma membrane increase the difficulty in the genetic engineering of thermophilic bacteria (Jiang et al., 2017). In order to rectify these issues, therefore, recent advancements in synthetic biology are expected to play a crucial role in the near future to construct better strains that would pave a way for consolidated bioprocessing (CBP) model to produce hydrolase, degrade polysaccharides, and ferment all derived sugars into ethanol (Lin and Xu, 2013). CBP, as a promising strategy for ethanol production, integrates enzyme production, saccharification, and fermentation into a one-step process. Strain *Clostridium thermocellum* ATCC 31924, with consolidated bioprocessing, significantly enhanced the cellulosic ethanol production up to 20% with crystalline cellulose as a substrate (Singh et al., 2018). A partially consolidated bioprocessing (PCBP) approach, including a non-isothermal simultaneous pretreatment and saccharification step using laccase and holocellulase by a co-fermentation of a mixture of *Ricinus communis, Saccharum officinarum*, and *Saccharum spontaneum* biomass, obtained a maximum ethanol concentration of 62.01 g/L (Althuri et al., 2017).

When compared with ethanol, biobutanol's lower volatility, lower heat of vaporization, higher viscosity, and higher energy density make it as a potential alternative to ethanol as a gasoline additive. In addition, butanol is more suitable for the existing oil transportation infrastructure due to its non-corrosiveness and poor hygroscopicity. The production and utilization of biological butanol has been studied for several decades, but there are still many challenges, including the low butanol production efficiency, toxicity to the production strain, multiple end-products, high energy consumption during recovery process of bio butanol, etc. (Wang et al., 2017). Therefore, it is an important goal to isolate more butanol producing extremophiles to further enhance the production level. Green Biologics company has developed a technology of producing bio butanol using corn raw materials by thermophilic Clostridium strains (Coker, 2016). In addition, *Thermoanaerobacterium, Pyrococcus*, and *Aeropyrum* were isolated, which showed great industrial production potential of biobutanol (De Vrije, 2002). In recent years, the metabolic engineering tools have already been used to for biobutanol production. In order to conquer the intractable gene operation of *Clostridium*, a series of methods were developed to improve the efficiency of electroporation, such as increasing the dissolution of cell membrane by adding solvent, weakening the cell wall by glycine or lysozyme, and optimizing the operation parameters of electroporation (Pyne et al., 2014). CRISPR-Cas tools have also been successfully applied to the genetic transformation of *Clostridium*. For example, the application of *Streptococcus pyogenes* II CRISPR-Cas9 system for the genome editing in *Clostridium acetobutylicum* DSM792^T^ has promoted it to utilize both glucose and xylose (Bruder et al., 2016). The TargeTron gene-editing technique, which depends on the mobility of group-II introns, was also found to be suitable for genetic manipulations in *Clostridium*. In addition, mesophilic-TargeTron and thermo-TargeTron technology was used for metabolic engineering and identification of functional genes (Wen et al., 2019).

In recent years, bio-jet fuel has attracted huge interest, and hence industries and scientists are currently working on technologies for converting biomass-based sustainable feedstocks into bio-jet fuels. Although most of the current bio-jet fuel technologies focus on chemistry methods, a considerable amount of research on the biotransformation of feedstocks into bio-jet fuel has also made a breakthrough in recent years. Researchers from the Chalmers University of Technology have developed a method to modify the enzyme fatty acid synthase for the synthesis of medium chain fatty acids and methyl ketones for jet fuel and biodiesel production (Zhu Z. et al., 2017). A research report has pointed out that the use of acetogenic bacteria that do not produce carbon dioxide by-products during fermentation can achieve high hydrocarbon biofuel production (330 L/t feedstock) (Crawford et al., 2016). It is one of the important directions of bio-jet fuel to isolate or construct microorganisms using synthetic biology approaches to convert sugar into alkanes efficiently. Cyanobacteria and oleaginous yeasts are considered to be the most suitable microorganisms for the production of alkanes (Jiménez-Díaz et al., 2017). It is a strategic priority to find out extremophiles with enhanced alkane production capabilities from extreme environmental niches could aid in the production of alkanes.

Oleaginous microorganisms, such as *Cryptococcus curvatus, Lipomyces starkeyi, Rhodosporidium toruloides, Rhodococcus opacus*, etc., can accumulate lipids using biomass as substrates (Tsigie et al., 2011). The accumulated lipids can be used as biodiesel feedstock for lignocellulosic biorefineries. Therefore, extremophiles could be a promising source to explore further for lipid production from the lignocellulosic hydrolysates as potential feedstock compliment (Poontawee et al., 2017). The use of lignocellulosic biomass to synthesize PHAs by halophilic microorganisms has recently attracted much attention. Although the yield is not high, it is still possible to obtain better results through genetic engineering of the target strains. Although the production of PHA from lignocellulose is still in the research stage, the result reported so far using this technology is promising as more and more extreme microorganisms gain an important position in biorefinery.

For the second generation of biorefining, enzymes are still the most proficient and expensive means. The use of extreme microorganisms and their enzymes to improve biorefinery processes has great potential. For example, the enzyme secreted by thermophilic bacteria exhibits higher activity and thermal stability at high temperatures, which was more effectively to depolymerize lignocellulose.

### Extremophiles and Lignin Valorization

Lignin is the most abundant natural aromatic compound in nature, accounting for 15 to 40% of the dry weight of plants. However, lignin was not fully utilized in the first-generation cellulose biofuel project. Although the research on lignin has a history of several decades, it still fails to develop an industrial technology that can realize the high-value utilization of lignin (Galkin and Samec, 2016). Recent studies have found that some microorganisms have evolved metabolic pathways, which can convert these aromatic substances into central intermediates through the “upper pathway,” and were then converted into central carbon metabolism through the “lower pathway.” This process is known as “Biological funneling.” “Biological funneling” provides a direct biological solution for the high-value utilization of lignin to overcome the heterogeneity problem in the lignin appreciation of modern biological refineries (Galkin and Samec, 2016). Screening ideal microorganisms and realizing the conversion of lignin flow to specific aromatic compounds are the hot research directions at present. Among them, extreme microorganisms and their enzymes are the focus of attention. Several extremophiles have shown their potential for high-value utilization of lignin, such as the halotolerant and alkalophilic bacterium *Bacillus ligniniphilus* L1, which is able to significantly degrade lignin at the optimal pH 9 and produces aromatic compounds, including vanillic acid, vanillin, etc. (Zhu D. et al., 2017). A thermophilic strain *Bacillus* sp. B1 was isolated from decayed wood bark can degrade cinnamic acid, ferulic acid, and coumaric acid into catechol, protocatechuic acid, and gentisic acid (Peng et al., 2003). In addition, a few thermo and halotolerant laccase were obtained from *Bacillus* sp. SS4, *Thermobifida fusca*, and *Trametes trogii*, and these laccase with their laccase-mediator systems (LMS) have potential applications for lignin valorization due to LMS being able to depolymerize lignin into low-molecular weight phenolics and aromatics (Chen et al., 2013; Christopher et al., 2014; Singh et al., 2019a; Yang et al., 2020). Therefore, the use of extremozymes to convert lignin into platform chemicals is one of the breakthrough applications of lignin valorization in the future.

### Recent Advancements in Extremozymes Discovery With Multi-Omics Approaches

In recent time, the term “omics,” including meta-genomics, meta-proteomics, meta-transcriptomics, or metabolomics, plays an important role in the discovery of new enzymes for biorefinery with improved activity. In addition, bioinformatics and algorithms play an irreplaceable role in design the *in situ* mutagenesis and gene shuffling to improve the stability of protein for potential industrial biorefinery purposes (Annamalai et al., 2016). These techniques have proved to be very useful for the development of extremozymes for biotechnology. Since more than 99% of microorganisms in the environment belongs to unculturable microorganisms, the current accessible techniques cannot obtain the target enzyme from the vast enzyme resource pool in nature. Therefore, “omics” technology has provided a powerful tool for the discovery of new enzymes from nature (Juerges and Hansjürgens, 2018).

In the last two decades, whole genome sequencing technology has been of great help to the understanding of the survival strategies of extremophiles in extreme environments. It promotes the understanding and application of the metabolic pathways, substrate biotransformation, transport mechanism, and enzymatic mechanism of extremophiles. Genomics research helps us to better understand the mechanism of robust enzymes and convey information about the three-dimensional structure of extreme enzymes. Based on the annotation and analysis of the genomic data of *Comamonas* SP 35, and combined with the metabolic analysis using GC-MS, the lignin degradation pathways were elucidated, and at least five metabolic pathways of lignin were speculated (Zhu D. et al., 2018). This is of practical significance for genetic transformation of chassis cells to achieve high-value utilization of lignin. Through the bioinformatics analysis of the whole genome sequence of psychrotolerant extremophile Pseudomonas sp. MPC6, the metabolism mechanism of toxic aromatic compounds was revealed, and the synthetase system of natural PHAs were identified. It was considered that Pseudomonas sp. MPC6 can be exploited as a biopolymer factory (Orellana-Saez et al., 2019). In recent years, the combination of computational and structure-based analysis with evolutionary driven methods (including directed evolution or synthetic biology) has been significantly enhanced by identifying novel extreme enzymes with high industrial application potential (Acevedo-Rocha et al., 2013; Ibrahim et al., 2016).

With the establishment of a large public database of genomic information, sequence-based methods, such as metagenomics, meta-transcriptomics, and meta-proteomics, have greatly increased the discovery of new biological systems (Naba et al., 2016; Caspi et al., 2019). Genomic data for more than 120 thermophilic bacteria are available in public database, and the genomes of *Pyrococcus, Anaerobranca, Thermotoga, Thermoplasma*, and *Thermus* genera have been studied in detail and provide enzymes appropriate for biorefinery industrial application (Counts et al., 2017). In addition, the “omics” technologies could resolve the bottleneck of research on unculturable microorganisms and find new high activity enzymes and metabolic pathways that can be used in biorefinery, which is the driving force to achieve sustainable biofuel production.

Transcriptomics approaches will help the study of the organism's total content of ribonucleic acid transcripts in a cell, including coding and non-coding RNAs, which can provide genome-wide data and information on gene functions to reveal molecular mechanisms related to specific biological processes. RNA sequencing technology has deepened researchers' understanding of RNA-based gene regulation and has become one of the areas of great concern in the post-genomic era. Transcriptome analysis of extremophiles is helpful to reveal the dynamic changes of gene expression in harsh environments and a more comprehensive understanding of the functional and regulating networks of microorganisms adapting to the living environments (Manzoni et al., 2016; Jorquera et al., 2019). A multi-omics analysis revealed that the thermal adaptation strategies of *Thermus filiformis*, including oxidative stress induced by high temperature, which lead to the inhibition of genes involved in glycolysis and tricarboxylic acid cycle; glucose metabolism is achieved mainly through the pentose phosphate pathway, or the glycolysis pathway, and the accumulation of oxaloacetic acid, α-ketoglutarate, and antioxidant enzymes related to free radical scavenging (Mandelli et al., 2017). Based on the transcriptome analysis of the digestive system of termites, it was revealed that more than 14 kinds of auxiliary oxidoreductases and glycosyl hydrolase genes (12 of them came from intestinal microorganisms) may be involved in the decomposition of lignin components and their redox networks during the process of biomass pretreatment. These finding suggested that the termites have a unique digestive system and provided new insights for biorefinery (Geng et al., 2018). In many studies, transcriptional engineering has been shown to be a powerful tool to improve recombinant bacteria. It will change the pH value, ion demand, and product specificity of wild-type strains and provide higher enzyme activity (Harman-Ware et al., 2017). The mutant α-amylase, as observed from the halophilic thermotolerant *Bacillus* strains cu-48, has brought better industrial applications (Bibra et al., 2017).

The proteomic analysis of extremophiles has paid more and more attention to the revealation of its special resistance to severe climate and environmental conditions. Proteome technology provides enough knowledge for exploring the survival mechanism of extremophiles and promoted the further application of extremophiles in the field of bioenergy (Blachowicz et al., 2019). A research study comparing the proteome of Bacillus ligniniphilus L1 with lignin as a substrate revealed that there are more than 30 kinds of upregulated enzymes involved in lignin degradation, such as peroxiredoxin, cytochrome oxidase, oxidoreductase, ferredoxin, etc. Many environmental response factors were also found, including repressor LexA, the DNA integrity scanning protein, the catabolite repression HPr-like protein, the central glycolytic genes regulator, and the transcriptional regulator, which positively regulates lignin as a substrate (Zhu D. et al., 2017). An LC–MS/MS shotgun proteomics analysis revealed that the obligate hydrocarbon-degrading psychrophile *Oleispira antarctica* RB-8 expressed a n-alkane oxidation pathway, including two alkane monooxygenases, two alcohol dehydrogenases, two aldehyde dehydrogenases, a fatty-acid-CoA ligase, and a fatty acid desaturase. When grown on tetradecane (n-C14), the synthesis of these proteins increased 3- to 21-fold compared with the control group (Gregson et al., 2020). However, compared to the existing methods, proteomics can better understand the characteristics of the enzyme, which are more advantageous for their industrial application. For example, the application of proteomics and gene-recombinant protein modification technologies helps to improve the characteristics of enzymes, such as thermal stability, higher activity, specificity, pH, solvent tolerance, etc. (Antikainen and Martin, 2005; Tesei et al., 2019). The information provided by genomics, proteomics, and transcriptome technologies can be used to identify new targets and metabolic engineering for the production of strains in the biorefinery industry. In this way, the metabolic regulations and the pathways can be extensively studied by multi-omics technologies to further improve the production and the performance of strains (Brugger et al., 2014; Furubayashi et al., 2014).

### Role Synthetic Biology in Extremophiles for Biorefinery

In the past two decades, synthetic biology has evolved as an important interdisciplinary research that integrates the genetic and metabolic engineering to customize genetic circuits to synthesize desired products using *E. coli* and *S. cerevisiae*. Henceforth, most of the synthetic biology research has been carried out using *E. coli* and *S. cerevisiae* (Adams et al., 2016). However, in recent years, researchers have begun to use several extremophiles, for example, *Deinococcus* spp, *Geobacillus* spp, *Halomonas* spp. *Pyrococcus* spp. *Thermococcus* spp, *Thermus* spp, etc., to develop industrially important strains. Synthetic biology approaches are handy in scheming organisms to thrive under modified/required conditions and to produce various bioactive molecules, which are of industrial and pharmaceutical importance through synthetic genetic circuits (Li et al., 2019). Synthesis of artemisinic acid, a precursor of artemisinin in *S. cerevisiae*, is one of the breaks through developments in synthetic biology maximizes the production and reduces the cost of artemisinin (Paddon et al., 2013). In case of biofuel and bioenergy, production of branched-chain higher alcohols from renewable resources using synthetic non-fermentative genetic circuit in *E. coli* (Atsumi et al., 2008) is a notable contribution of synthetic biology. Though significant development has been achieved in terms of lignin valorization, several bottle neck conditions have arisen that are attributable to high recalcitrance and the heterogeneous nature of lignin (Liu et al., 2019). High-value bioconversion of lignin is a three-step process that includes depolymerization, aromatics degradation, and synthesis of the required end product. Hence, system-level identification of mechanisms and pathways using integrated (meta)genomics, (meta)transcriptomics, (meta)proteomics, (meta) sectretomics, and metabolomics approaches has paved a way to create lignin conversion synthetic genetic circuits, and metabolically engineered ligninolytic strains are amenable hosts for high-value lignin bioconversion. In addition, metabolic engineering and conventional adaptation methods are useful in development of strains with an ability to grow well in the presence of high concentration of aromatic compounds aroused from lignin depolymerization, which often hampers the growth of microorganisms. Lin et al. have developed a *Pseudomonas putida* strain (A514) capable of producing PHA using insoluble kraft lignin as the sole carbon source through peroxidase-based depolymerization, the metabolism of aromatic byproducts, followed by rechanneling of β-oxidation products (Lin et al., 2016). Metabolic versatility and stability of *Thermoacidophilic Sulfolobus* species has attracted researchers to use it as one of the promising platforms for synthetic biology and metabolic engineering (Schocke et al., 2019). The availability of whole genome sequences, the construction of marker-free in-frame deletion mutants, and the homologs expression of proteins via ectopic integration of foreign genes in *Sulfolobus acidocaldarius* and *Sulfolobus islandicus* allows the modulation of the regulatory mechanisms and rechanneling of metabolic pathways to improve the production (Wagner et al., 2014).

The *S. cerevisiae* INVSc1 strain, equipped with a synthetic genetic circuit containing heat shock protein and superoxide dismutase from Thermus thermophiles HB8 and *Thermoanaerobacter tengcongensis* MB4, respectively, can grow well at 42°C and produces significantly more ethanol than its wild type (Sun et al., 2017). It is noteworthy to mention that alcohol dehydrogenases from extremophiles have been proved to be an excellent catalyst to produce butanol using cell-free systems (Karim et al., 2019). Wu et al. (2017) have developed a method that includes chemical depolymerization of alkali lignin into vanillin and syringate and their bioconversion into cis, cis-muconic acid, and pyrogallol, respectively, by means of metabolically engineered *E. coli* strains. In an investigation, Kohlstedt et al. (2018) metabolically engineered *P. putida* KT2440 MA-9 to produce cis and cis-muconic acid by using hydrothermally depolymerized lignin aromatics as a source, which is hydrogenated to form adipic acid and finally polymerized into nylon.

Deletion of vanillin dehydrogenase from the industrially important strains has been shown to enhance the production of vanillin from lignocellulose biomass (Linger et al., 2014). Alternatively, a thermo regulated-genetic system, i.e., the heterologous expression of two key enzymes, such as feruloyl-CoA synthetase (Fcs) and enoyl-CoA hydratase/aldolase (Ech) of thermophilic actinomycete *Amycolatopsis thermoflava* N1165 in *E. coli*, can also be used. This system allows *E. coli* to produce vanillyl alcohol using ferulic acid as a source at 30°C and subsequent conversion of vanillyl alcohol into vanillin at 50°C by the enzymatic activities of Fcs and Ech (Ni et al., 2018a). The abovementioned synthetic pathway is, however, effective in terms of function for the synthesis of vanillin from lignin-derived aromatic compounds affected by the requirement of high energy. Hence, phenolic acid decarboxylase (Pad) and aromatic dioxygenase (Ado), a Coenzyme-Free enzymatic pathway, was introduced into *E. coli*, which. in turn. allows it to the conversion of lignin derived aromatic compounds to vanillin and 4-vinylphenol (Ni et al., 2018b). Utilization of renewable sources for the production of biofuels and bioenergy is a much-needed industrial sector to cope with global warming and draining of fossil fuels. Recent advancements in multi-omics technologies, the discovery of highly efficient lignin-degrading enzymes, the integration of physical, chemical, and biological methods in lignin depolymerization, and the need for economically viable and eco-friendly production of biofuel and bioenergy necessitates researchers to engineer industrially important microorganisms through a synthetic biology approach.

## Conclusion and Future Perspectives

In the past two decades, many countries around the world have invested a lot of money into basic research and the technological development of first- and second-generation biofuels and bioenergy, respectively. In order to deal with the reduction of fossil energy reserves and the requirements of environmental protection, many biorefinery methods have been developed by researchers, but most of them stay in the laboratory or pilot stage; only a few have entered in industries for successful production. Though biofuel industries based on different technical routes have been built around the world, they still have to manage huge limitations in the form of production cost, maintenance, and technology development issues as well as an insufficient maturity of technology, high cost, and the lack of commercial competitiveness. Reports have shown that the pretreatment takes about 40% of the total biomass processing cost (Sindhu et al., 2016). At present, one of the biggest problems in biorefinery is that it has not yet achieved the full utilization of lignin, cellulose, and hemicellulose. One of the key bottlenecks in the green revolution is difficulties associated with biodegradation of lignin. These problems have to be addressed in a scientific, eco-friendly, and cost-effective way, which in turn will boost the industrial processes for sustainable production of biofuel.

Extremophiles and extremozymes bring the dawn of success for biorefinery owing to their specificity, robustness in action, and high tolerance to the adverse conditions of the biorefinery process. It is expected that more extreme bacteria and their enzymes/proteins will provide more competitive chassis cells and catalysts for the production of biofuels in an efficient way. Of course, the realization of all these goals necessitates the assistance of available traditional and modern cutting edge molecular biological tools. However, the production cost of extreme enzyme is still much higher than that of conventional enzyme preparation. For example, 8,000 units of endo-1,4-β-xylanase cost 177 euros from *Thermotoga maritima* but only 149 euros from *Trichoderma viride* (Ebaid et al., 2019). In addition, the high-density fermentation of halophilic bacteria requires more than 10% salt content in the culture medium, which requires better corrosion resistance of the production equipment as well as the high cost of environmental protection brought by hyper-saline wastewater. Therefore, the chassis cells derived from extremophiles were transformed by synthetic biological methods, the highly active extremophiles were screened based on histochemistry technology, and new metabolic pathways were explored to optimize the technical route. The development of artificial intelligence and nanomaterial technology brings the innovation of production equipment and improves the competitiveness of bioenergy plants.

## Author Contributions

DZ conceived and designed the manuscript. JS supervised the manuscript. DZ and WA wrote the manuscript. FA, SS, and BD revised the manuscript.

## Conflict of Interest

The authors declare that the research was conducted in the absence of any commercial or financial relationships that could be construed as a potential conflict of interest.
